# OD32 Single Nucleotide Polymorphism Marker Analysis In Donor-Derived Cell-Free DNA For Solid Organ Transplantation (Next-Generation Sequencing): Systematic Review

**DOI:** 10.1017/S0266462324001636

**Published:** 2025-01-07

**Authors:** Yoojin Kim, Jiae Im, Wurlsook Lee, Jieun Choi

## Abstract

**Introduction:**

Single nucleotide polymorphism marker analysis in donor-derived cell-free (dd-cf ) DNA for solid organ transplantation is a technique for post-transplantation monitoring, including early detection of injury to the transplanted organ, signs of infection, and treatment decision-making, by measuring dd-cf-DNAs as a percentage of the total cf-DNAs in the patient’s body in donors and recipients of kidney, heart, lung, or liver transplants.

**Methods:**

The assessments were performed via a systematic review. Searching five databases (KoreaMed, Ovid MEDLINE, Ovid Embase, and Cochrane) yielded 1,619 related studies. Two reviewers independently assessed the quality of these studies using a Scottish Intercollegiate Guidelines Network checklist, and the assessment results were described based on the results of the quality appraisal and level of evidence.

**Results:**

The population of the included studies was patients who underwent kidney, heart, lung, or liver transplantation. Medical outcomes in kidney transplantation patients were reported in 15 studies. The index test was reported to have an area under curve (AUC) of 0.68 to 0.99 and a sensitivity of 0.24 to 1.00. Four studies reported effectiveness data for the index test for lung transplantation patients. The diagnostic accuracy of the index test for acute cell-mediated rejection was reported have an AUC of 0.72. Sensitivity was reported as 0.44 and specificity as 0.80 for heart transplant patients, and sensitivity as 0.73 to 1.00 and specificity as 0.60 to 0.95 for liver transplant patients.

**Conclusions:**

This is a safe and effective technique for post-transplantation monitoring, including the early detection of injury to the transplanted organ and signs of infection, and for treatment decision-making, by measuring dd-cf-DNA as a percentage of total cf-DNA in the patient’s body in recipients and donors of kidney, heart, lung, or liver transplantation (level of evidence: C).

